# Editorial: Advancing Theory of Suicide and Non-Suicidal Self-Injury

**DOI:** 10.3389/fpsyg.2021.780029

**Published:** 2021-11-22

**Authors:** Kathryn Jane Gardner, E. David Klonsky, Edward A. Selby

**Affiliations:** ^1^School of Psychology, University of Central Lancashire, Preston, United Kingdom; ^2^Department of Psychology, University of British Columbia, Vancouver, BC, Canada; ^3^Department of Psychology, Rutgers University, Piscataway, NJ, United States

**Keywords:** non-suicidal and suicidal self-injurious thoughts and behaviours, self-harm, suicide, suicidal behaviour, theory, non-suicidal self-injury

The past decade has seen an explosion of empirical studies devoted to better understanding of self-harm by focusing both on non-suicidal self-injury (NSSI) and on suicide risk outcomes (including non-fatal suicidal thoughts and behaviour), and their key distinctions. Both NSSI and suicide are important public health issues that are associated with psychological distress and impairment (Klonsky et al., 2003; Selby et al., 2012; Brunner et al., 2014; Victor and Klonsky, 2014; Eskin et al., 2016) and significant economic impact worldwide (Sinclair et al., 2011; Florence et al., 2015; Shepard et al., 2015; Kinchin et al., 2017; Doran and Kinchin, 2020; Tsiachristas et al., 2020). Self-injury (including NSSI and past suicidal behaviour) is also an essential risk factor for future suicidal behaviour (Hamza et al., 2012; Ribeiro et al., 2016; Castellví et al., 2017), with suicide being among the top 10 leading causes of death in eastern Europe, central Europe, western Europe, central Asia, Australasia, southern Latin America, and high-income North America (Naghavi, 2019). It is imperative that we continue to develop and refine evidence-based psychological theory so we can better understand, prevent, and treat both NSSI and suicidal behaviour.

In this special issue we therefore showcase papers that advance conceptual and theoretical understandings of NSSI and/or suicide, or which address critical open questions about the nature and operationalisation of these behaviours that must be answered before we put theory to the test and address conceptual gaps in the field. The collection of articles, submitted from across Europe, America, Canada, and Asia, includes: three review papers that inform theory and future research on understanding NSSI and suicide risk outcomes; one conceptual analysis of suicide-specific syndromes; and seven empirical studies. The studies address: operationalisation and stability of NSSI and suicidal behaviour; the relationship between NSSI and suicidality; the co-occurrence of NSSI with other disorders and dysregulated behaviours; and mechanisms, mediators, and moderators underlying NSSI and suicidality.

The first primary theme pertains to “fluctuations in non-suicidal self-injurious and suicidal behaviour.” Repetition of NSSI is common, especially among adolescents (Brunner et al., 2007; Hawton et al., 2012; Howe-Martin et al., 2012), leading researchers to identify the mechanisms that might explain why the behaviour is frequent returned to. Repetition of NSSI, for example, is defined in the Diagnostic and Statistical Manual of Mental Disorders (DSM-5; American Psychiatric Association, 2013) as five or more NSSI experiences, and Lee and Hyun's study in University students meeting this threshold suggests that repeat self-injury could be underpinned by general difficulties in behavioural control. Like several other studies in this special issue, this was a cross-sectional one, paving the way for future prospective studies that can identify whether these effects persist over time and simultaneously explain fluctuations in NSSI and suicidality. Indeed, a major takeaway from the special section was that consistency of self-reported NSSI and suicidal behaviour shouldn't be taken for granted. Daukantaite et al., for example, found in psychiatric inpatients that NSSI frequency dropped substantially after the initial assessment (including originally reported lifetime NSSI frequency). Fluctuations were also found in a second potential suicide risk variable, NSSI functions. In our own work with individuals in the community (Gardner et al.), we similarly found changes in functions over time. In addition, intrapersonal (e.g., affect regulatory) functions were a prospective risk factor for repeated future self-harm, and *potentially* suicide attempts. Changes in suicide risk factors were also explored in a University student sample by Law and Anestis; the authors identified changes in suicide capability, which has previously been thought of as a more static trait. Research focusing on suicidal behaviour, especially NSSI, and associated risk variables should be mindful of the variability in patient recalls. Likewise, fluctuations in suicidal ideation and behaviour are likely to dovetail with clinical case conceptualizations of suicidal behaviour as representing acute risk or crisis syndromes characterised by repeated suicidal behaviour (as outlined in the conceptual paper by Voros et al.).

There was also a key theme of better understanding individual perceptual experience in suicidal and NSSI behaviour, and the better we can develop a conceptual theory of mind for such individuals, the better we can tailor our interventions to their needs. In samples of University students Hamza et al. found that exposure to recent stressful experiences was associated with perceived heightened emotional reactivity, which in turn, increased the risk of NSSI; while the Mettler et al. study indicated that those with a history of NSSI may have less accurate perceptions of their emotions and emotion management abilities. The Pollak et al. study of community adolescents identified that a propensity towards imagining more positive future events, especially those less realistic and achievable, was predictive of suicide ideation, possibly because of feeling defeated or trapped by those ambitions. Mental imagery and awareness of future consequences can differentiate individuals who are suicide ideators from attempters, and this is central to the theory outlined by Macintyre et al. which integrates a number of specific concepts into three principles of control, conflict, and awareness.

The social context within which perceptual experiences occur is just as important to understand, and this has also been a focus of empirical studies in the field. The social determinants of behaviour are central to the review by Mueller et al.'s which draws on sociological theories of death by suicide as a basis for recommending that psychology theories place increased emphasis on the external social world that extends beyond individuals' perceptions of it. The theoretical paper by Shafti et al.'s  positions social context within their cognitive-emotional model of “*dual harm*,” a concept that accounts for the co-occurrence of non-suicidal self-harm and aggression and can be explained by common proximal and distal risk factors within the individual (e.g., personality) or their environment (e.g., socially adverse situations or events).

The final theme identified in the special issue pertained to placing suicidal behaviours in the greater context of an individual's life and other aspects of mental health. So often suicidal or NSSI behaviours are studied outside the context of comorbid diagnoses that can dramatically affect an individual's symptom severity or prognosis. For example, in their graphical network examination of self-injury symptoms relative to borderline personality disorder symptoms among adolescence, Buelens et al. found that self-injury conceptualised as a disorder was functionally distinct from borderline personality disorder symptoms. There were additional key symptoms that “bridged” the two symptom clusters, namely loneliness, impulsivity, separation anxiety, and self-injury thoughts and affect, which may serve as key early intervention targets in adolescents. Furthermore, suicidal behaviours do not exist isolated from an individual's existential life, experience, and purpose, and yet so much of our assessment and treatment approach is focused solely on symptoms and acute distress. In their study on childhood abuse and spirituality amongst outpatients with suicide attempts, Tae and Chae found that spirituality offered a protective effect against the negative outcomes of childhood abuse and associated suicide risk. There may be room to improve assessments and interventions to incorporate measurement of patient spiritual beliefs or other factors relevant to making meaning out of a difficult life. Doing so is likely to help us reach patients in more personal and possibly more durable ways.

Therefore, as research on suicide risk outcomes and NSSI behaviour continues, we encourage consideration of the key themes identified in this special issue. It is important to recognise suicide risk outcomes and NSSI, and even some key risk factors, as constantly evolving phenomena. Likewise, individual perceptions and the accuracy of those perceptions can influence risk for these behaviours, especially under key social contexts. Finally, suicidal and NSSI behaviour exist as just parts of an individual's life, and better understanding the broader life context surrounding these behaviours may be essential to tailoring and improving current intervention strategies.

## Author Contributions

KG and ES led on the initial draft of the editorial. All authors have contributed and approved the final version.

## Conflict of Interest

The authors declare that the research was conducted in the absence of any commercial or financial relationships that could be construed as a potential conflict of interest.

## Publisher's Note

All claims expressed in this article are solely those of the authors and do not necessarily represent those of their affiliated organizations, or those of the publisher, the editors and the reviewers. Any product that may be evaluated in this article, or claim that may be made by its manufacturer, is not guaranteed or endorsed by the publisher.
